# Body fat variation and redistribution across different stages of life measured by dual-energy x-ray absorptiometry

**DOI:** 10.7189/jogh.14.04247

**Published:** 2024-11-15

**Authors:** Hongbo Dong, Hong Cheng, Jingfan Xiong, Li Liu, Yiwen Huang, Xinying Shan, Hongmin Fan, Xi Wang, Xia Wang, Pei Xiao, Fangfang Chen, Jie Mi

**Affiliations:** 1 Center for Noncommunicable Disease Management, Beijing Children's Hospital, Capital Medical University, National Center for Children's Health, Beijing, China; 2 Department of Epidemiology, Capital Institute of Pediatrics, Beijing, China; 3 Child and Adolescent Chronic Disease Prevention and Control Department, Shenzhen Center for Chronic Disease Control, Shenzhen, China; 4 School of Public Health, Guangdong Pharmaceutical University, Guangzhou, China; 5 Child Healthcare Center, Children’s Hospital, Capital Institute of Pediatrics, Beijing, China; 6 North China University of Science and Technology, Hebei, Tangshan, China

## Abstract

**Background:**

The global obesity epidemic of all ages has increased the demand for accurate management of body fat in each stage of life. The primary aim of this study was to provide reference centiles of body fat indices for Chinese children and adults and compare those with ethnicities from USA using dual-energy x-ray absorptiometry (DXA).

**Methods:**

The samples were drawn from two nationwide cross-sectional surveys of China Body Composition Life-course Study (2013–2023) and the US National Health and Nutrition Examination Survey (2011–2018). Age- and sex-specific centile curves were generated for a set of fat measurements, including total fat mass (FM), fat mass index (FMI), body fat percentage (BF%), trunk-to-leg fat ratio (TLR), android-to-gynoid fat ratio (AGR) and visceral-to-subcutaneous fat ratio (VSR), using the general additive model for location scale and shape method.

**Results:**

The age-related variations from childhood to adulthood were generally similar among Chinese, Non-Hispanic Whites, Non-Hispanic Blacks and Mexican American population, with distinct levels across races and ethnicities. For whole-body fat (FM, FMI, and BF%), Mexican American population consistently presented the highest levels before 40 years old, followed by Non-Hispanic White, Non-Hispanic Black and Chinese individuals. For central fats indices, although the TLR and AGR levels in Chinese males were second to Mexican American counterparts in most stages of life, the VSR was much higher in Chinese than other races and ethnicities from eight years old.

**Conclusions:**

DXA-based centiles for body fat quantity and distribution in Chinese population aged 3–60 years old were presented, and their differences with other race and ethnicity were noted across periods from early childhood to middle adulthood. Our findings will promote age-, sex- and ethnic-specific assessment of life-course body fat and obesity-related risks in clinical practice.

The obesity epidemic is one of the greatest public health challenges of our time, with the prevalence increasing at an alarming rate in many countries [1,2]. According to the Global Burden of Disease study estimates for 2021, China and the USA were among the top five countries with the highest absolute burden of obesity [3]. Although obesity is closely related to a variety of comorbidities, such as diabetes, cardiovascular diseases and cancers, the commonly used anthropometric indicator, body mass index (BMI), does not comprehensively capture the true nature of obesity, i.e. abnormal or excessive fat accumulation or their heterogeneous health impacts [4]. In the era of precision medicine, the focus of obesity management has been increasingly switched from weight to individualised direct body fat measures [5–7].

With the advent of body composition analysis techniques, body fat can be directly assessed by a range of methods, such as magnetic resonance imaging, computed tomography, dual-energy x-ray absorptiometry (DXA) and bioelectrical impedance [8]. Among these methods, DXA has been recognised as a potent tool for quantifying total and regional body fat mass due to its low radiation exposure, high precision, and strong clinical feasibility [9]. Several studies have investigated the age-related adiposity changes based on the DXA method [10–13], including those from China and the USA. But most of them were piecemeal, with limited range of ages and divergent device manufacturers. Given the inconsistency of their analysis algorithms, it is difficult to understand the development of adiposity from a life-course perspective. Moreover, it has been proven that Chinese individuals have greater amount of unfavourable central adipose tissue than their Western counterparts [14,15]. However, little is known about the age-related variations in these disparities over the lifespan.

To address this issue, we expanded our earlier paediatric work into adulthood, to explore the developmental patterns of whole-body fat quantity and regional fat distribution by constructing a comprehensive set of sex- and age-specific body fat references derived from DXA among the Chinese population. Additionally, to reveal the potential racial differences in body fat changes and redistribution with age, we further compared our results with individuals of other races and ethnicities from the National Health and Nutrition Examination Survey (NHANES).

## METHODS

### Study population

Our study included two cross-sectional data sets from the China Body Composition Life-course (BCL) Study and the NHANES (2011–2018). The China BCL study is a nationwide, ongoing population-based cross-sectional study involving nine urban areas (i.e. Beijing, Tianjin, Tangshan, Changchun, Jinan and Yinchuan, Shanghai, Chongqing and Guangzhou) in China. This study was designed to determine patterns of body composition change over the life course of the well-nourished Chinese population. The first round of the BCL study, also known as the China Child and Adolescent Cardiovascular Health study, which was conducted from 2013 to 2019. A sample of 14 600 children and adolescents aged 3–18 years were recruited from selected schools in eight cities; the detailed sampling procedures have been described in detail elsewhere [16,17]. In 2021, the BCL study began to enrol healthy volunteers who were young- or middle-aged adults from the staff of selected universities, research institutions and hospitals in three cities. Until August 2023, 5297 adults aged over 18 years participated and underwent DXA scans. In total, 18 334 individuals aged 3–60 years with valid whole-body DXA scans were collected in the first and second rounds of the BCL study. To ensure that the body fat change patterns reflect the biological variation in a disease-free population, we further excluded individuals who had chronic metabolic diseases (diabetes mellitus, kidney disease, heart disease or thyroid disease), or who were currently taking medications (n = 631), or who had unremovable objects or body parts outside of the scan region (n = 40). Finally, a total of 17 691 individuals (8578 males and 9113 females) aged 3–80 years were eligible for analysis of age-related body fat changes in the Chinese population.

The National Health and Nutrition Examination Survey is a well-known programme of studies designed to assess the health and nutritional status of the children and adults in USA. Dual-energy x-ray absorptiometry scans were taken for subjects older than eight years beginning in 1999 [18]. To compare body fat levels with Chinese population during contemporary period, data from 17 833 USA subjects who underwent whole-body DXA scans between 2011 and 2018 were extracted from the NHANES online public database.

Data for this study included children and adults form USA who self-reported as Non-Hispanic White, Non-Hispanic Black and Mexican American individuals. We excluded individuals who self-reported as other Hispanic and other race (including multi-racial) (n = 5128); those with chronic diseases or who were taking medications (n = 1230); and those with unremovable objects or body parts outside of the scan region (n = 419); 11 802 individuals were included in the analysis. As the age range of the individuals included in the BCL study (3–60 y) was wider than that included in the NHANES (8–59 y), the data of the Chinese population (n = 17 691) were used to explore the developmental patterns of body fat quantity and distribution. For further comparison analysis of ethnic differences, 3184 subjects under eight years or over 59 years old from the BCL study were excluded, and the data of 14 445 Chinese and 11 802 Non-Hispanic Whites, Non-Hispanic Blacks and Mexican Americans were finally included. The flowchart of inclusion and exclusion is shown in Figure S1 in the **Online Supplementary Document**.

### Anthropometric measurements

The data were collected by trained staff according to similar procedures and collection methods in the BCL study and the NHANES. Anthropometric measurements were conducted on the same day before performing DXA scans. Height was measured without shoes by a stadiometer with a fixed vertical backboard and an adjustable headpiece. Weight was measured in lightweight clothing with barefoot on a calibrated digital scale. Readings for weight and height were taken to the nearest 0.1 kg and 0.1 cm, respectively, and BMI was then calculated as weight (in kg) divided by the square of height (in m^2^). The participants were classified into four BMI groups as underweight, normal weight, overweight and obese, using the cut-offs for age and sex recommended by the International Obesity Task Force [19].

### Whole-body DXA scan

In both the BCL study and the NHANES, whole-body DXA scans were obtained using Hologic Discovery fan-beam densitometers according to standard operating methods [20]. The total and regional fat mass of the trunk, leg, android and gynoid, as well as abdominal visceral and subcutaneous fat were then automatically quantified by the APEX software (Hologic, Inc., Waltham, MA, USA). The details concerning the delimitation of regions of body in DXA scans has been described previously [21]. In brief, the trunk region is composed by neck, chest, abdomen and pelvis; the leg region includes the area below the lower border of the trunk; the android region is the area between the last thoracic rib and the upper part of iliac wings; the gynoid region is below the android region, representing the gluteofemoral area approximately between the head of the femur and the midthigh; the abdominal visceral and subcutaneous fat were analysed within the android region, which was a five cm thick region placed above the iliac crest at a level that approximately coincided with the fourth lumbar vertebrae.

The fat mass index (FMI) was calculated as total fat mass (kg) / height (m^2^). Body fat percentage (BF%) was calculated as total body fat mass (kg) / total body mass (kg) × 100%. The fat percentages in the trunk, leg, android, gynoid, visceral and subcutaneous regions were respectively calculated in a similar manner as that used for the BF%. The trunk-to-leg fat ratio (TLR) was calculated as trunk fat mass / leg fat mass, and the android-to-gynoid fat ratio (AGR) was calculated as android fat mass / gynoid fat mass. The visceral-to-subcutaneous fat ratio (VSR) was calculated as the visceral fat area / subcutaneous fat area.

### Other covariates

Standardised questionnaires were conducted by face-to-face interview in both BCL study and the NHANES among subjects over eight years old. Data with similar questions on demographics (age, sex), family income and lifestyle behaviours (physical activity, sugar-sweetened beverages, night sleeping duration) were obtained. Family income was classified based on the poverty-to-income ratio (PIR), an index derived by dividing family annual income by the established poverty level, accounting for household size and year of assessment. We categorised the family income as low-income level (PIR<1.3), middle-income level (PIR = 1–3.5), and high-income level (PIR>3.5). Subjects with frequencies of moderate or vigorous physical activities less than 60 minutes daily were defined as being physically inactive. The intake of sugary drinks, including sodas, fruit drinks, energy drinks, sports drinks and sweetened bottled waters, was categorised as frequent if individuals were consumed daily. The night sleep duration was classified into sufficient group and insufficient group according to the National Sleep Foundation’s sleep time duration recommendations [22].

### Statistical analysis

Continuous variables were expressed as mean with standard deviation (SD), and categorical variables were expressed as frequency with percentage. Comparisons of differences in characteristics between sexes were performed using χ^2^ tests for categorical variables, and analysis of covariance adjusted for age for continuous variables.

Sex- and age-specific curves were generated for each body fat indicator according to age by using The Generalized Additive Model for Location Scale and Shape [23]. This approach allows linear and/or nonlinear and/or nonparametric modelling of the explanatory variables using distributions with up to four parameters that are commonly represented by μ for location, σ for scale and ν and τ for shape. The distribution of Normal, Box-Cox Cole and Green, Box-Cox Power Exponential, and Box-Cox-t were tried to fit the observed distributions, and the additive functions of linear, cubic splines, penalised splines, loess and fractional polynomials were used to model the influence of age on parameters of the considered distribution. The final models were chosen by the Bayesian information criterion. The reference values of body fat parameter centiles (5th, 25th, 50th, 75th and 95th) were computed at specific ages for Chinese. The age-, sex- and ethnic-specific body fat centile curves of total FM, FMI, BF%, TLR, AGR and VSR were also modelled in Non-Hispanic White, Non-Hispanic Black and Mexican American individuals. The 50th centile of each body fat indicator was used to illustrate age-related variation in Chinese and all three races and ethnicities from the USA. Furthermore, ethnic differences across different stages of life were also compared using analysis of covariance adjusted for age, socioeconomic status, physical activity, sugary drink consumption and sleep duration, to rule out potential confounders.

All the statistical analyses were conducted using *R* software, version 4.1.1 (R Core Team 2021, Vienna, Austria). *P*-values <0.05 (two-sided) were considered statistically significant.

## RESULTS

The descriptive characteristics of the study population are shown in **Table 1**. For the Chinese population, the overall prevalence rate of BMI-defined overweight and obesity was 27.3%, with rates of 19.7 and 28.9% in those aged 3–7 years and over eight years, respectively. The latter was much lower than the Non-Hispanic White individuals (58.3%), Non-Hispanic Black individuals (63.1%), and Mexican American individuals (70.7%). In each stage of lives from childhood to adulthood, statistically greater rate of BMI-defined overweight and obesity were found in males than females for Chinese population, which were generally similar between sexes for ethnicities from the USA. Moreover, males presented statistically lower rate of being physically inactive but more frequent intake of sugary drinks than their female counterparts.

**Table 1:** Characteristics of the study population

Characteristics	Chinese individuals		Non-Hispanic White individuals†		Non-Hispanic Black individuals†		Mexican American individuals†	
	**Male**	**Female**	**Male**	**Female**	**Male**	**Female**	**Male**	**Female**
Age group, n (%)								
*3–7y*	1658 (19.3)	1526 (16.7)	NA	NA	NA	NA	NA	NA
*8–11 y*	1786 (20.8)	1628 (17.9)	393 (14.5)	371 (15.7)	360 (18.3)	342 (18.9)	274 (18.5)	313 (21.3)
*12–19 y*	3519 (41.0)	3591 (39.4)	601 (22.2)	515 (21.8)	541 (27.5)	452 (25.0)	447 (30.1)	434 (29.6)
*20–39 y*	1242 (14.5)	1473 (16.2)	868 (32.0)	784 (33.1)	542 (27.6)	450 (24.9)	412 (27.8)	355 (24.2)
*40–60y*	373 (4.3)	895 (9.8)	848 (31.3)	697 (29.4)	522 (26.6)	565 (31.2)	351 (23.7)	365 (24.9)
Height (cm), x̄ (SD)	154.1 (22.4)	149.4 (17.7)*	169.9 (14.9)	159.9 (10.7)*	168.2 (15.0)	159.2 (10.6)*	164.0 (14.0)	153.7 (10.1)*
Weight (kg) x̄ (SD)	51.4 (22.0)	45.9 (16.1)*	77.0 (26.5)	68.1 (23.0)*	74.9 (27.8)	72.9 (26.3)*	73.9 (25.1)	64.9 (22.2)*
BMI (kg/m^2^), x̄ (SD)	20.5 (4.7)	19.8 (4.0)*	25.9 (6.8)	26.2 (7.6)	25.7 (7.3)	28.2 (8.8)*	26.8 (6.8)	27.0 (7.6)
BMI status, n (%)								
*Underweight*	226 (2.6)	390 (4.3)	38 (1.4)	43 (1.8)	27 (1.4)	32 (1.8)	11 (0.7)	16 (1.1)
*Normal*	5388 (62.8)	6868 (75.4)	1043 (38.6)	990 (41.9)	788 (40.2)	541 (30.0)	397 (26.9)	438 (30.0)
*Overweight*	1924 (22.4)	1434 (15.7)	843 (31.2)	605 (25.6)	565 (28.8)	448 (24.8)	518 (35.1)	449 (30.7)*
*Obese*	1040 (12.1)	421 (4.6)*	781 (28.9)	726 (30.7)*	582 (29.7)	783 (43.4)*	550 (37.3)	559 (38.2)
Frequent intake of sugary drinks, n (%)	1512 (27.1)	1507 (24.4)*	1627 (62.8)	1191 (52.5)*	1314 (71.8)	1203 (70.5)	1001 (72.1)	927 (67.2)*
Physically inactive, n (%)	4428 (77.0)	5333 (88.6)*	950 (38.4)	1080 (52.7)*	732 (41.2)	802 (53.7)*	530 (40.0)	680 (55.4)*
Insufficient night sleep, n (%)	2160 (32.6)	2493 (34.3)*	690 (34.6)	483 (27.8)*	592 (45.4)	537 (43.5)	305 (31.1)	263 (28.1)
Family income, n (%)								
*Low*	1736 (25.0)	1867 (24.7)*	808 (31.1)	697 (30.7)	747 (42.0)	751 (45.4)	620 (47.2)	683 (52.7)*
*Middle*	2264 (32.6)	2311 (30.6)	924 (35.6)	795 (35.0)	682 (38.3)	601 (36.3)	528 (40.2)	454 (35.0)
*High*	2936 (42.3)	3379 (44.7)	864 (33.3)	779 (34.3)	350 (19.7)	302 (18.3)	166 (12.6)	160 (12.3)

BMI – body mass index, SD – standard deviation.

*Statistically significant between sexes, *P* < 0.05.

†The data for the included USA population were eligible from eight years of age, and the information for sleep duration were eligible from participants aged 16 and older.

### Body fat development spanning from age 3 to age 60

**Figure 1:**
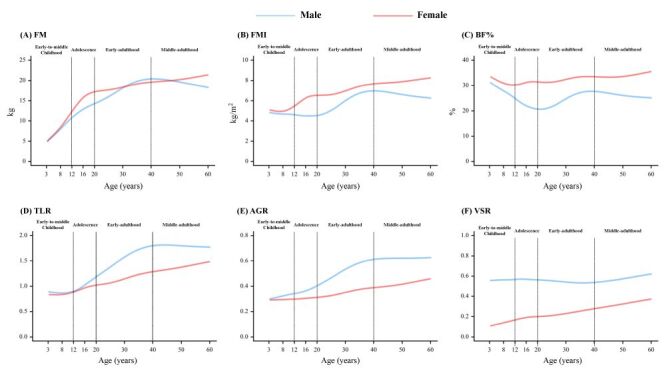
The 50th centile curves for body fat measures of Chinese males and females aged 3–60 years. **Panel A.** FM. **Panel B.** FMI. **Panel C.** BF%. **Panel D.** TLR. **Panel E.** AGR. **Panel F.** VSR. AGR – android-to-gynoid fat ratio, BF% – body fat percentage, FM – fat mass, FMI – fat mass index, TLR – trunk-to-leg fat ratio, VSR – visceral-to-subcutaneous fat ratio.

The curves for body fat quantity and distribution measures during the six decades of life among the Chinese population are illustrated in **Figure 1** and Figure S2 in the **Online Supplementary Document**. In general, males sustainably displayed lower levels of whole-body fat (i.e. FM, FMI, and BF%) but higher levels of central fat (relative percentage of fat mass around trunk, android and visceral regions) and a greater tendency of central distribution (i.e. TLR, AGR, and VSR) than females at most stages of life from three to 60 years of age, except for surpassing FM levels in female at approximately 30–50 years of age. Moreover, unlike the constant ascending trends in the TLR, AGR and relative percentage of central fat around the trunk and android regions, age-related fluctuations were demonstrated for whole-body fat, peripheral fat percentage and VSR.

Specifically, FMI and BF% decreased with age in both boys and girls during early childhood, and divergent patterns were subsequently depicted between sexes. In contrast to the continuing upward trend of FMI and BF% in females, the FMI of males decreased to the nadir at the end of adolescence, followed by a dramatic increase to its peak at approximately 40 years. The age-related patterns of BF% were similar to those of FMI, with sharper decreases during childhood and adolescence. In addition, slight declines were illustrated for all whole-body fat indices from 40 years onward in males. For the leg- and gynoid-deposited fat in both sexes, the relative percentage increased during early- and middle-childhood and peaked at approximately 12 years for leg fat percentage and approximately 20 years for gynoid fat percentage, followed by consistent decreases afterwards. The opposite trends were presented for relative fat percentage and distribution around abdominal regions, which shifted from downward to upward after 16 years of age for visceral and subcutaneous fat percentage in both sexes and after 35 years for VSR in males. For the VSR in females, steady growth was observed across different stages of life. The smoothed age-specific centile values for each body fat measure are provided in Tables S1–S12 in the **Online Supplementary Document**.

### Racial and ethnic disparities in age-related changes of body fat

**Figure 2:**
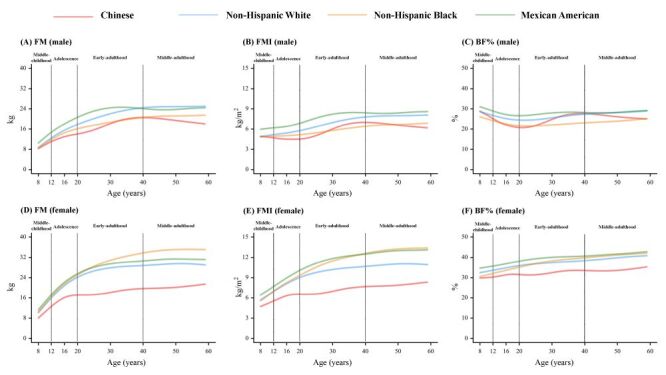
Comparisons of the 50th centile curves for body fat measures among Chinese, the Non-Hispanic White, Non-Hispanic Black and Mexican American males and females aged 8–59 years. **Panel A.** FM in males. **Panel B.** FMI in males. **Panel C.** BF% in males. **Panel D.** FM in females. **Panel E.** FMI in females. **Panel F.** BF% in females. BF% – body fat percentage, FM – fat mass, FMI – fat mass index.

**Figure 3:**
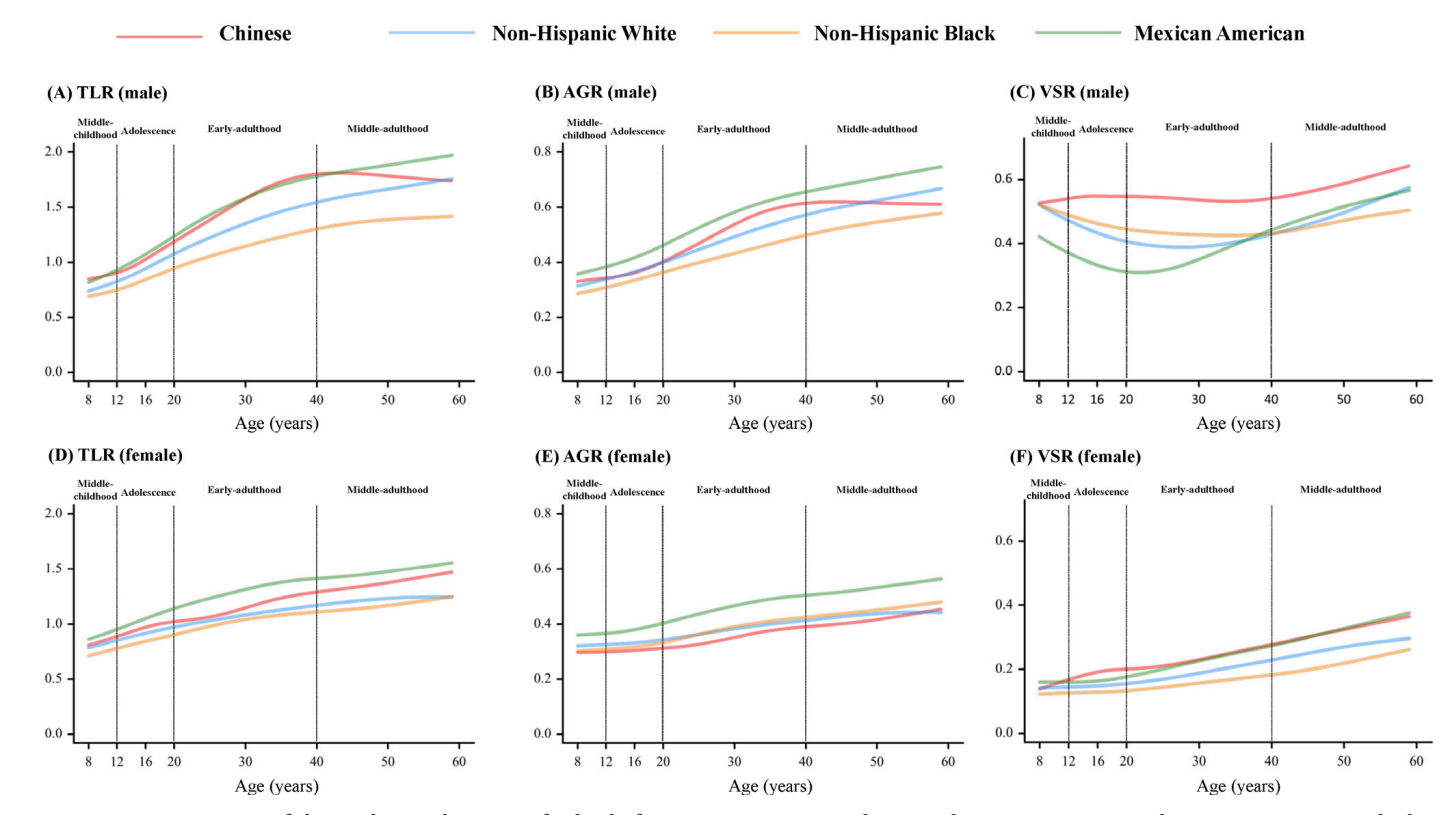
Comparisons of the 50th centile curves for body fat measures among Chinese, the Non-Hispanic White, Non-Hispanic Black and Mexican American males and females aged 8–59 years. **Panel A.** TLR in males. **Panel B.** AGR in males. **Panel C.** VSR in males. **Panel D.** TLR in females. **Panel E.** AGR in females. **Panel F.** VSR in females. AGR – android-to-gynoid fat ratio, TLR – trunk-to-leg fat ratio, VSR – visceral-to-subcutaneous fat ratio.

The comparison of body fat curves among Chinese, the Non-Hispanic White, Non-Hispanic Black and Mexican American individuals aged 8–59 years are shown in **Figure 2** and **Figure 3**. Despite the generally ascending trends of whole-body fat, a non-dropping profile of FMI was exhibited in adolescent males of all three races and ethnicities in the USA population, but not in the Chinese. In addition, the relative ethnic rankings of most fat measures were stable in the USA population (Mexican American>Non-Hispanic Whites>Non-Hispanic Blacks), except for the FM in females (Non-Hispanic Blacks>Mexican American>Non-Hispanic Whites) and VSR before 40 years (Non-Hispanic Blacks>Non-Hispanic Whites>Mexican American). However, the relative rankings of body fat between Chinese and other ethnicities varied by indictor, age and sex. For whole-body fat indices, lowest levels were found in Chinese females for all three indices and in Chinese males for FM. The FMI and BF% of Chinese males surpassed the Non-Hispanic Black males after 30 years of age. For central fat indices, the AGR and TLR levels in Chinese were close to those in whites but second to those in Mexican American population in most stages of life; and the highest VSR level was found in Chinese after eight years old in males and 12 years old in females.

## DISCUSSION

Dual-energy x-ray absorptiometry is widely considered as a valid method for measuring body fat and have several advantages over other techniques. Unlike the assumption of constant body density in anthropometric- and body density-based methods, total body fat mass determined by DXA only relies on the specific x-ray attenuation properties through lipid (triglycerides, phospholipid membranes, etc.). It gives the promise of accuracy over a wide range of ages, ethnicities and body types [24]. Moreover, DXA can measure regional body fat, including abdominal visceral fat, providing additional information for further risk stratification. It has been shown that an increase AGR is associated with higher risks of multiple cardiometabolic risk factors, such as hypertension, impaired glucose tolerance, insulin resistance, and dyslipidaemia [25,26]. Additionally, higher TLR indicated with greater risk of lipodystrophy, especially for HIV infected patients [27]. The clinical importance of visceral adipose tissue is widely recognised, which is predictor of several cardiovascular diseases and mortality [28]. However, methodologies like hydro-densitometry, bioelectrical impedance, and isotope dilution are limited in their ability to determine body fat distribution. Furthermore, DXA is more accessible and comfortable for patients than other body composition techniques. For DXA with the last generation of fan-beam densitometers, it only took three minutes for a whole-body scan, with much less time than magnetic resonance imaging, and less dose of radiation compared with computed tomography. It should be noted that DXA might underestimate whole-body fat mass compared with magnetic resonance imaging or computed tomography, and could not perform on pregnant women due to the x-ray exposure. Even though, DXA was recommended as the reference technique for the body fat assessment [29]. Based on this reference method, the quantity and distribution of body fat were accurately characterised among population of wide range of ages and race and ethnicities. This study would contribute knowledge about racial disparities in body fat at different life-course stages.

### Age-related changes in body fat quantity and distribution

Understanding the developmental patterns of body fat is fundamental to interpretating body fat measurements. Unfortunately, previous studies are often scattered and lack holistic knowledge on the complete age-related changes in body fat quantity and distribution. To date, only two studies from North America have evaluated adiposity changes from childhood to adulthood [18,30]. However, both studies missed the age of adiposity rebound, which was suggested to be a critical stage for adiposity development and is particularly associated with later health. In current research, we extended the study population to three years of age and found steady decreases in whole-body fat indices throughout entire early childhood, followed by the occurrence of adiposity rebound at eight years. Although this timing was 2–3 years later than that derived from BMI measures [31], it is close to that derived from DXA-based cohort studies [13], indicating altered growth patterns of adiposity from BMI as early as early childhood.

It is well-known that adipose tissue undergoes critical changes in abundance, distribution, and action with aging, leading to a decrease in subcutaneous adipose tissue and the accrual of visceral adipose tissue [32,33]. However, the exact timing for age-associated adiposity redistribution remains unclear. In this study, we noted a clear reversal shift in growth patterns between VSR and whole-body fat around 35–40 years in the male population. In further comparative analyses, similar patterns were also observed in males of all three races and ethnicities from the USA. Thus, the quantity of whole-body fat maybe unable to discern age-related adiposity redistribution in the abdominal regions among males. Given the mounting evidence for the additive value of the VSR in the morbidity and mortality prediction beyond the FMI or BF% [34–37], the necessities of monitoring the distribution of abdominal fat after middle adulthood should be emphasised.

### Sex differences in age-related changes of body fat

Consistent with the findings of previous studies, we found greater quantity of body fat but lower centrally distributed adiposity in females than in males. Additionally, age-associated sex disparities were depicted, which was largest during adolescence for FMI and BF% but around 35–40 years old for AGR and TLR. Both human and rodent studies have demonstrated the impact of sex hormones (oestrogens and testosterone) on the accumulation and distribution of adipose tissue [38]. Oestrogens have been proved to modulate lipolysis and lipogenesis via oestrogen receptors on adipose tissue, thereby facilitating adipose tissue expansion and inhibiting lipid storage in visceral adipose tissue. Testosterone, on the contrary, inhibits lipid uptake in adipocytes and directs body fat accrual away from the gluteofemoral depot. The oestrogen and testosterone levels are both varied by age: increases occur at 11 years in boys and at nine years in girls [39]; the decline in testosterone starts after the age of 20–30 years, while the loss of oestrogens begins after the menopause around 50–60 years [40,41]. Therefore, age-related changes in sex hormones may help explain the sexual dimorphism in body fat development, which is beyond the scope of our investigation and warrants further research.

### Racial and ethnic disparities in body fat development

Apart from ageing and sex, race and ethnicity are another known determinant of changes in body fat quantity and distribution. In current study, we demonstrated almost the lowest level of body fat mass but the highest level of relative central distribution of whole-body and abdominal adiposity in Chinese than those of other races and ethnicities. The underlying mechanisms for the racial and ethnic disparities in body fat development are complex of social constructs, and environmental, social, behavioural, and nutritional factors. Differences in the frequencies of genes from different population ancestry may also contribute to varied body fat shapes and related prevalence of diseases. In this study, we noted the intake of sugary drinks were more frequent in USA Mexican American and Non-Hispanic Black individuals. Greater proportions of being physically active were found in Non-Hispanic Blacks and Non-Hispanic Whites than Chinese in each stage of life. After controlling these potential confounders, racial and ethnic disparities in body fat development were still pronounced (Tables S13–14 in the **Online Supplementary Document**), indicating the role of genetic background in body fat accumulation and redistribution. Together, these findings highlight the importance of lifelong assessment of age-, sex- and ethnic-specific body fat

### Strengths and limitations

The major strength of the study is that we first illustrated the development of body fat quantity and distribution in different ethnicities from childhood to adulthood based on large samples (n >20 000) of data from nations with the largest population of obese individuals. Both data sets were obtained from the same DXA manufacturers and contemporary population, guaranteeing the comparable results across ethnicities. This study has several limitations. First, the current results are based on cross-sectional data. Although cross-sectional data are commonly used to detect age-related changes in anthropometric indices with the assumption of generally stable socioeconomic growth background, a more appropriate (but less practical) assessment of an individual’s body fat changes should be based on data generated from a longitudinal sample. Second, our data from BCL study is not nationally representative, and the results may not be generalisable to the entire Chinese population. Third, our participants may have limited relevance to Chinese immigrants from the USA or multiracial populations, which remains in need of further research. Fourth, several behavioural aspects of diet and early life factors that may impact the body fat development were not included as covariates for analysing ethnic differences.

## CONCLUSIONS

In the present study, we characterised the development of body fat quantity and distribution in the Chinese population from early childhood to middle adulthood, and compared their differences with three races and ethnicities from the USA. Given the highly obesogenic environment of both the USA and China, our findings highlight the need for lifelong application of age-, sex- and ethnic-specific body fat assessment in clinical practice, which will assist accurate management of obesity and further risk stratification.

## Additional material


Online Supplementary Document
