# Supplementary material for: Body fat variation and redistribution across different stages of life measured by dual-energy x-ray absorptiometry
Source: J Glob Health. 2024 Nov 15;14:04247. doi: 10.7189/jogh.14.04247 (PMC11565468; doi:10.7189/jogh.14.04247)
Supplement: Online Supplementary Document [file jogh-14-04247-s001.pdf]

Body fat variation and redistribution across different stages of life using dual-energy X-ray absorptiometry

TABLE OF CONTENTS

Figure S1. The flow chart of inclusion and exclusion.

Figure S2. The 50th centile curves for trunk and leg fat percentage (a), android and gynoid fat percentage (b), and visceral and subcutaneous fat percentage (c) in Chinese males and females aged 3 to 60 years.

Table S1. Centiles for total fat mass (FM, in kg) by age in Chinese males and females aged 3-60 years.

Table S2. Centiles for total fat mass index (FMI, in kg/m^2) by age in Chinese males and females aged 3-60 years.

Table S3. Centiles for total body fat percentage (BF%) by age in Chinese males and females aged 3-60 years.

Table S4. Centiles for trunk-to-leg fat ratio (TLR) by age in Chinese males and females aged 3-60 years.

Table S5. Centiles for android-to-gynoid fat ratio (AGR) by age in Chinese males and females aged 3-60 years.

Table S6. Centiles for visceral-to-subcutaneous fat ratio (VSR) by age in Chinese males and females aged 3-60 years.

Table S7. Centiles for trunk fat percentage by age in Chinese males and females aged 3-60 years.

Table S7. Centiles for trunk fat percentage by age in Chinese males and females aged 3-60 years.

Table S8. Centiles for leg fat percentage by age in Chinese males and females aged 3-60 years.

Table S9. Centiles for android fat percentage by age in Chinese males and females aged 3-60 years.

Table 10. Centiles for gynoid fat percentage by age in Chinese males and females aged 3-60 years.

Table S11. Centiles for visceral fat percentage by age in Chinese males and females aged 3-60 years.

Table 12. Centiles for subcutaneous fat percentage by age in Chinese males and females aged 3-60 years.

Table S13. Covariates-adjusted total fat mass (FM), fat mass index (FMI) and body fat percentage (BF%) among Chinese, US non-Hispanic White, non-Hispanic Black and Mexican American across different stages of life.

Table S14. Covariates-adjusted trunk-to-leg fat ratio (TLR), android-to-gynoid fat ratio (AGR), and visceral-to-subcutaneous fat ratio (VSR) among Chinese, US non-Hispanic White, non-Hispanic Black and Mexican American across different stages of life.

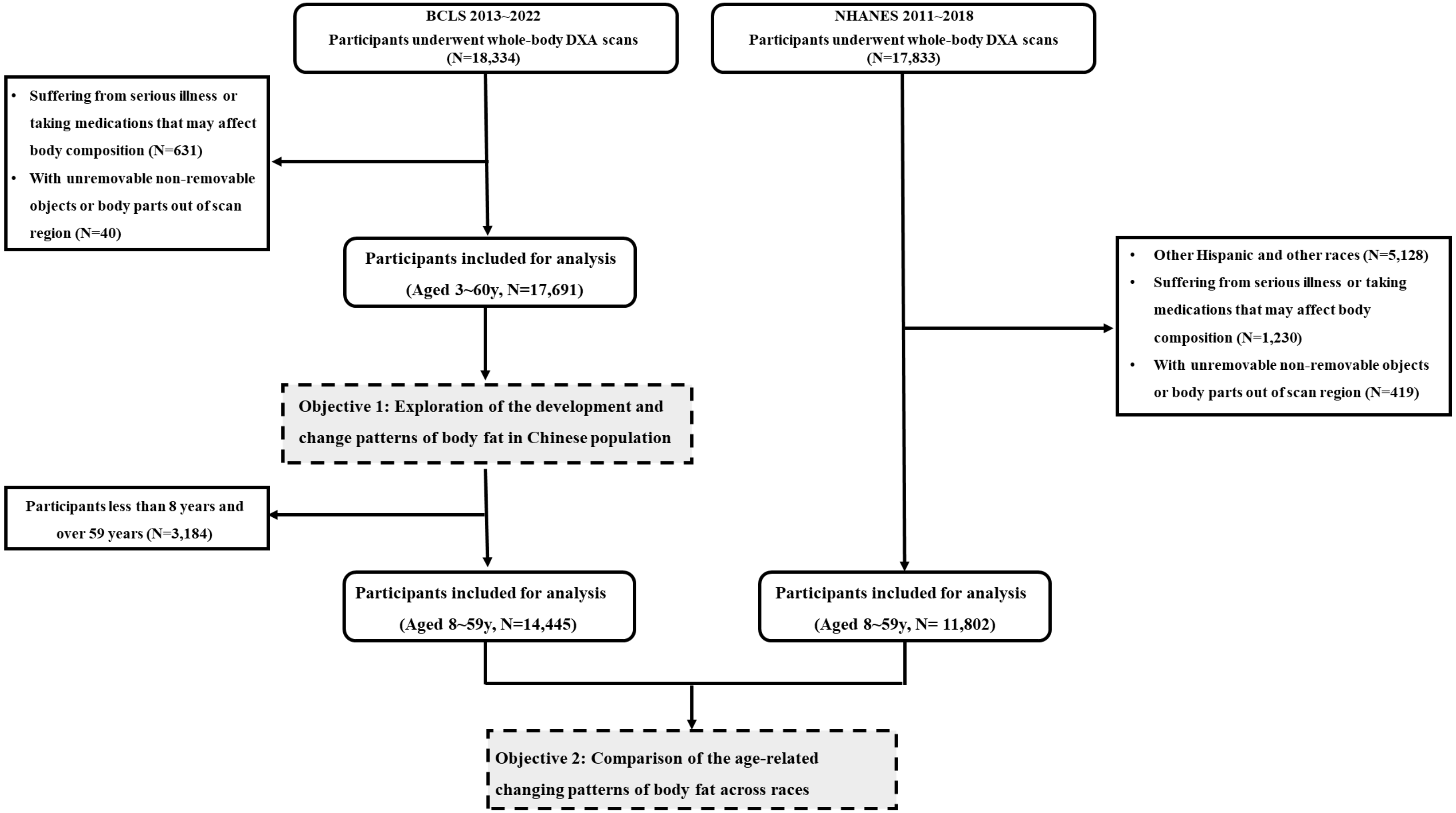

Figure S1. The flow chart of inclusion and exclusion

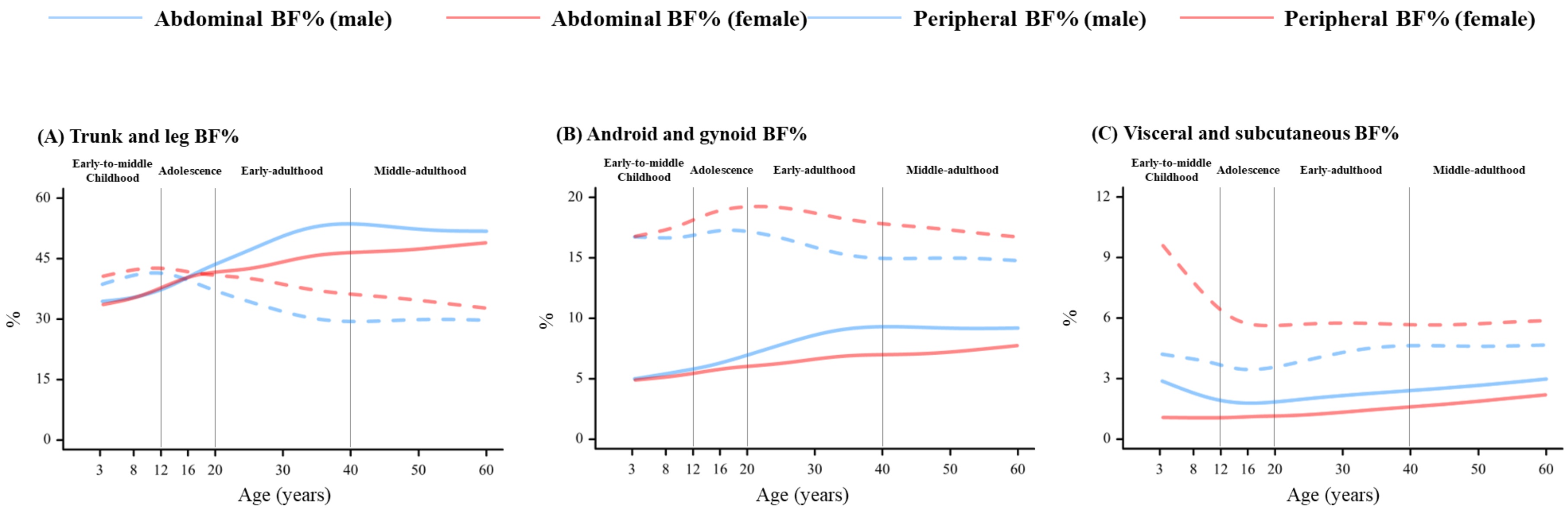

Figure S2. The 50th centile curves for regional body fat percentage in Chinese males and females aged 3 to 60 years. **Panel A.** Trunk and leg BF% TLR. **Panel B.** Android and gynoid BF%. **Panel C.** Visceral and subcutaneous BF%. BF% – body fat percentage.

Table S1. Centiles for total fat mass (FM, in kg) by age in Chinese males and females aged 3-60 years.

Age, yrs	Male					Female				
	5th	25th	50th	75th	95th	5th	25th	50th	75th	95th
3	3.2	4.1	4.7	5.5	7.7	3.2	4.2	4.8	5.6	7.5
4	3.5	4.5	5.4	6.5	9.2	3.6	4.7	5.5	6.5	8.8
5	3.7	4.9	6.0	7.5	10.9	3.9	5.2	6.2	7.5	10.3
6	4.0	5.3	6.6	8.6	12.7	4.2	5.6	6.9	8.5	11.9
7	4.3	5.6	7.3	9.8	14.9	4.5	6.1	7.6	9.6	13.6
8	4.5	6.0	8.0	11.1	17.2	4.9	6.7	8.5	10.8	15.5
9	4.8	6.4	8.7	12.4	19.7	5.3	7.3	9.4	12.1	17.5
10	5.1	6.8	9.4	13.7	22.2	5.8	8.0	10.3	13.5	19.6
11	5.4	7.3	10.1	14.9	24.5	6.4	8.8	11.3	14.8	21.5
12	5.7	7.7	10.8	16.0	26.4	7.0	9.6	12.3	16.0	23.3
13	6.0	8.1	11.4	16.9	27.9	7.6	10.5	13.3	17.2	24.8
14	6.3	8.6	12.0	17.6	29.1	8.3	11.3	14.3	18.2	26.1
15	6.6	9.0	12.5	18.2	30.0	8.9	12.1	15.1	19.0	27.1
16	6.9	9.4	12.9	18.7	30.8	9.4	12.8	15.8	19.7	27.9
17	7.1	9.7	13.3	19.1	31.5	9.9	13.3	16.3	20.2	28.5
18	7.3	10.1	13.7	19.5	32.2	10.2	13.7	16.7	20.6	29.0
19	7.5	10.3	14.0	19.8	32.9	10.4	13.9	17.0	20.9	29.4
20	7.7	10.6	14.3	20.1	33.5	10.5	14.1	17.2	21.2	29.7
25	8.8	12.3	16.0	21.8	36.3	10.7	14.3	17.7	22.1	31.0
30	10.5	14.4	18.2	23.7	37.4	11.2	14.8	18.4	23.0	31.8
35	12.1	16.1	19.8	24.9	37.3	12.0	15.6	19.1	23.6	32.0
40	12.8	17.0	20.4	24.9	36.7	12.5	16.2	19.6	23.9	32.0
45	12.8	17.0	20.2	24.3	35.8	12.7	16.6	19.9	24.0	32.3
50	12.4	16.6	19.6	23.6	34.9	13.0	17.0	20.2	24.1	32.7
55	11.9	15.9	19.0	23.1	34.0	13.5	17.7	20.8	24.5	33.1
60	11.5	15.0	18.3	22.8	33.1	14.1	18.4	21.4	24.9	33.6

Table S2. Centiles for total fat mass index (FMI, in kg/m2) by age in Chinese males and females aged 3-60 years.

Age, yrs	Male					Female				
	5th	25th	50th	75th	95th	5th	25th	50th	75th	95th
3	3.4	4.3	4.8	5.5	7.4	3.6	4.5	5.1	5.8	7.5
4	3.3	4.1	4.8	5.7	7.7	3.5	4.4	5.0	5.9	7.6
5	3.1	4.0	4.8	5.8	8.0	3.3	4.2	5.0	5.9	7.8
6	3.0	3.8	4.7	5.9	8.4	3.2	4.1	5.0	6.0	8.0
7	2.9	3.7	4.7	6.1	8.8	3.1	4.1	4.9	6.1	8.2
8	2.8	3.6	4.7	6.3	9.3	3.1	4.0	5.0	6.2	8.5
9	2.7	3.5	4.7	6.5	9.7	3.0	4.0	5.0	6.4	8.8
10	2.6	3.5	4.7	6.6	10.2	3.1	4.1	5.1	6.5	9.1
11	2.6	3.4	4.7	6.7	10.5	3.1	4.2	5.3	6.8	9.5
12	2.5	3.4	4.6	6.7	10.6	3.2	4.3	5.5	7.0	9.8
13	2.5	3.3	4.6	6.7	10.7	3.4	4.5	5.7	7.2	10.1
14	2.5	3.3	4.5	6.7	10.7	3.5	4.7	5.9	7.4	10.4
15	2.5	3.3	4.5	6.6	10.6	3.7	4.9	6.1	7.6	10.6
16	2.5	3.3	4.5	6.6	10.5	3.8	5.1	6.3	7.7	10.8
17	2.5	3.3	4.5	6.5	10.4	3.9	5.2	6.4	7.8	10.9
18	2.5	3.3	4.5	6.4	10.4	4.0	5.3	6.5	7.9	11.0
19	2.5	3.3	4.5	6.4	10.3	4.1	5.4	6.5	8.0	11.0
20	2.5	3.4	4.5	6.4	10.3	4.1	5.4	6.5	8.0	11.0
25	2.9	3.9	5.1	6.8	11.0	4.1	5.4	6.6	8.2	11.3
30	3.6	4.8	6.0	7.7	12.0	4.3	5.7	7.0	8.7	11.9
35	4.2	5.6	6.8	8.4	12.5	4.7	6.1	7.4	9.1	12.4
40	4.4	5.9	7.0	8.4	12.4	4.9	6.4	7.7	9.3	12.5
45	4.4	5.9	6.9	8.1	11.9	5.0	6.5	7.8	9.3	12.5
50	4.3	5.7	6.6	7.8	11.5	5.2	6.7	7.9	9.3	12.6
55	4.2	5.4	6.4	7.7	11.1	5.3	6.9	8.1	9.4	12.6
60	4.1	5.2	6.3	7.6	10.7	5.6	7.2	8.3	9.6	12.7

Table S3. Centiles for total body fat percentage (BF%) by age in Chinese males and females aged 3-60 years.

Age, yrs	Male					Female				
	5th	25th	50th	75th	95th	5th	25th	50th	75th	95th
3	24.8	28.6	31.4	34.5	40.1	25.9	30.6	33.7	36.9	42.1
4	23.7	27.6	30.7	34.3	40.2	25.0	29.7	33.1	36.6	41.8
5	22.6	26.5	30.0	34.1	40.4	24.1	28.9	32.5	36.2	41.6
6	21.5	25.4	29.3	33.9	40.6	23.2	28.1	31.9	35.8	41.3
7	20.4	24.4	28.6	33.8	40.8	22.4	27.4	31.4	35.5	41.1
8	19.4	23.4	28.0	33.6	41.1	21.8	26.8	30.9	35.3	40.9
9	18.4	22.4	27.3	33.4	41.3	21.3	26.3	30.6	35.0	40.8
10	17.6	21.5	26.6	33.0	41.3	20.9	26.0	30.3	34.9	40.6
11	16.7	20.7	25.8	32.4	41.0	20.8	25.8	30.2	34.8	40.6
12	16.0	19.8	25.0	31.7	40.3	20.8	25.9	30.2	34.8	40.6
13	15.3	19.1	24.1	30.8	39.4	21.0	26.1	30.4	34.9	40.7
14	14.7	18.4	23.3	29.8	38.3	21.3	26.4	30.6	35.1	40.9
15	14.2	17.9	22.6	28.9	37.3	21.6	26.7	30.9	35.3	41.1
16	13.9	17.5	22.1	28.1	36.3	21.9	27.1	31.2	35.5	41.4
17	13.6	17.1	21.6	27.4	35.5	22.1	27.3	31.4	35.7	41.6
18	13.4	16.9	21.2	26.8	34.8	22.2	27.5	31.5	35.7	41.7
19	13.2	16.7	20.9	26.3	34.2	22.2	27.5	31.5	35.7	41.7
20	13.1	16.7	20.7	26.0	33.8	22.1	27.4	31.4	35.7	41.6
25	14.1	17.9	21.7	26.6	34.4	22.0	27.1	31.4	35.8	41.6
30	16.9	21.0	24.8	29.4	37.2	23.1	28.1	32.5	36.9	42.7
35	19.3	23.7	27.2	31.3	39.1	24.4	29.4	33.5	37.7	43.3
40	20.0	24.7	27.7	31.3	39.0	24.6	29.6	33.5	37.6	43.1
45	19.5	24.1	27.0	30.3	38.0	24.5	29.4	33.3	37.4	42.9
50	18.8	23.2	26.1	29.5	37.0	24.7	29.7	33.6	37.6	43.2
55	18.3	22.4	25.5	29.1	36.2	25.7	30.9	34.4	38.1	43.9
60	18.1	21.8	25.1	29.1	35.5	27.0	32.5	35.5	38.7	44.7

Table S4. Centiles for trunk-to-leg fat ratio (TLR) by age in Chinese males and females aged 3-60 years.

Age, yrs	Male					Female				
	5th	25th	50th	75th	95th	5th	25th	50th	75th	95th
3	0.63	0.79	0.89	1.01	1.35	0.64	0.75	0.83	0.93	1.10
4	0.65	0.79	0.88	1.00	1.27	0.64	0.75	0.83	0.93	1.10
5	0.66	0.78	0.88	0.99	1.22	0.64	0.74	0.83	0.93	1.10
6	0.66	0.78	0.87	0.98	1.20	0.64	0.74	0.83	0.93	1.11
7	0.66	0.77	0.87	0.98	1.19	0.64	0.74	0.83	0.93	1.11
8	0.66	0.77	0.86	0.97	1.18	0.64	0.75	0.83	0.93	1.12
9	0.66	0.77	0.87	0.98	1.19	0.64	0.75	0.84	0.94	1.13
10	0.67	0.78	0.87	0.99	1.20	0.65	0.76	0.85	0.96	1.15
11	0.67	0.79	0.88	1.00	1.21	0.66	0.77	0.87	0.98	1.17
12	0.69	0.80	0.90	1.02	1.23	0.67	0.79	0.88	1.00	1.20
13	0.71	0.82	0.92	1.04	1.26	0.69	0.81	0.90	1.02	1.23
14	0.73	0.85	0.95	1.07	1.29	0.70	0.82	0.93	1.04	1.26
15	0.76	0.88	0.98	1.11	1.33	0.71	0.84	0.95	1.07	1.30
16	0.79	0.92	1.02	1.15	1.38	0.73	0.86	0.97	1.09	1.33
17	0.82	0.95	1.06	1.19	1.44	0.74	0.87	0.98	1.12	1.36
18	0.84	0.99	1.10	1.24	1.51	0.75	0.89	1.00	1.13	1.38
19	0.86	1.02	1.14	1.29	1.59	0.76	0.90	1.01	1.15	1.40
20	0.88	1.06	1.18	1.34	1.67	0.76	0.90	1.02	1.16	1.42
25	0.99	1.21	1.38	1.58	2.07	0.79	0.94	1.07	1.22	1.50
30	1.13	1.37	1.57	1.82	2.34	0.83	1.00	1.15	1.32	1.64
35	1.25	1.50	1.72	2.00	2.54	0.88	1.07	1.23	1.42	1.79
40	1.31	1.57	1.80	2.08	2.62	0.91	1.12	1.29	1.50	1.91
45	1.34	1.59	1.81	2.08	2.60	0.93	1.14	1.33	1.56	2.01
50	1.34	1.59	1.80	2.06	2.56	0.95	1.18	1.38	1.63	2.12
55	1.32	1.57	1.78	2.04	2.55	0.97	1.22	1.43	1.70	2.25
60	1.27	1.55	1.77	2.04	2.66	0.99	1.25	1.49	1.78	2.39

Table S5. Centiles for android-to-gynoid fat ratio (AGR) by age in Chinese males and females aged 3-60 years.

Age, yrs	Male					Female				
	5th	25th	50th	75th	95th	5th	25th	50th	75th	95th
3	0.24	0.27	0.30	0.33	0.38	0.23	0.26	0.29	0.32	0.38
4	0.24	0.27	0.30	0.34	0.40	0.23	0.26	0.29	0.33	0.39
5	0.24	0.28	0.31	0.34	0.41	0.23	0.26	0.29	0.33	0.40
6	0.24	0.28	0.31	0.35	0.43	0.22	0.26	0.29	0.33	0.40
7	0.24	0.28	0.32	0.36	0.45	0.22	0.26	0.29	0.34	0.41
8	0.24	0.28	0.32	0.37	0.46	0.22	0.26	0.29	0.34	0.42
9	0.24	0.29	0.33	0.38	0.48	0.22	0.26	0.30	0.34	0.42
10	0.24	0.29	0.33	0.39	0.50	0.22	0.26	0.30	0.34	0.43
11	0.24	0.29	0.34	0.39	0.51	0.22	0.26	0.30	0.34	0.43
12	0.24	0.30	0.34	0.40	0.52	0.22	0.26	0.30	0.34	0.43
13	0.25	0.30	0.35	0.40	0.52	0.22	0.26	0.30	0.35	0.43
14	0.25	0.30	0.35	0.41	0.52	0.22	0.26	0.30	0.35	0.43
15	0.26	0.31	0.36	0.41	0.53	0.22	0.27	0.30	0.35	0.44
16	0.26	0.32	0.36	0.42	0.54	0.22	0.27	0.30	0.35	0.44
17	0.27	0.33	0.37	0.43	0.55	0.23	0.27	0.31	0.35	0.44
18	0.28	0.33	0.38	0.44	0.56	0.23	0.27	0.31	0.35	0.44
19	0.28	0.34	0.39	0.45	0.58	0.23	0.27	0.31	0.36	0.44
20	0.29	0.35	0.40	0.47	0.60	0.23	0.27	0.31	0.36	0.44
25	0.34	0.41	0.47	0.54	0.70	0.24	0.29	0.33	0.38	0.47
30	0.38	0.47	0.53	0.62	0.81	0.25	0.30	0.35	0.41	0.53
35	0.42	0.51	0.59	0.68	0.89	0.26	0.32	0.38	0.44	0.58
40	0.44	0.53	0.61	0.72	0.92	0.27	0.33	0.39	0.46	0.61
45	0.45	0.54	0.62	0.72	0.93	0.28	0.34	0.40	0.47	0.62
50	0.45	0.54	0.62	0.72	0.93	0.29	0.36	0.42	0.49	0.64
55	0.45	0.54	0.62	0.72	0.94	0.31	0.38	0.44	0.51	0.66
60	0.43	0.55	0.63	0.73	1.00	0.33	0.40	0.46	0.53	0.68

Table S6. Centiles for visceral-to-subcutaneous fat ratio (VSR) by age in Chinese males and females aged 3-60 years.

Age, yrs	Male					Female				
	5th	25th	50th	75th	95th	5th	25th	50th	75th	95th
3	0.20	0.38	0.56	0.73	0.90	0.02	0.07	0.11	0.14	0.22
4	0.20	0.38	0.56	0.73	0.90	0.03	0.08	0.11	0.15	0.22
5	0.20	0.39	0.56	0.73	0.90	0.03	0.08	0.12	0.16	0.23
6	0.21	0.39	0.56	0.73	0.90	0.04	0.09	0.13	0.17	0.24
7	0.21	0.39	0.56	0.73	0.90	0.04	0.09	0.13	0.17	0.25
8	0.21	0.39	0.56	0.73	0.90	0.05	0.10	0.14	0.18	0.25
9	0.22	0.39	0.56	0.73	0.89	0.05	0.11	0.15	0.19	0.26
10	0.22	0.40	0.56	0.73	0.89	0.06	0.12	0.15	0.19	0.26
11	0.22	0.40	0.57	0.73	0.89	0.07	0.12	0.16	0.20	0.27
12	0.22	0.40	0.57	0.73	0.89	0.08	0.13	0.17	0.21	0.27
13	0.23	0.40	0.57	0.73	0.89	0.08	0.14	0.17	0.21	0.28
14	0.23	0.40	0.57	0.73	0.89	0.09	0.14	0.18	0.22	0.28
15	0.23	0.41	0.57	0.73	0.89	0.10	0.15	0.19	0.22	0.29
16	0.24	0.41	0.57	0.73	0.89	0.10	0.16	0.19	0.23	0.29
17	0.24	0.41	0.57	0.72	0.88	0.10	0.16	0.19	0.23	0.30
18	0.24	0.41	0.57	0.72	0.88	0.11	0.16	0.20	0.24	0.30
19	0.24	0.41	0.56	0.72	0.87	0.11	0.16	0.20	0.24	0.30
20	0.24	0.41	0.56	0.72	0.87	0.11	0.16	0.20	0.24	0.30
25	0.25	0.41	0.55	0.70	0.84	0.11	0.17	0.21	0.25	0.33
30	0.26	0.40	0.54	0.68	0.81	0.12	0.18	0.23	0.28	0.36
35	0.26	0.40	0.53	0.66	0.79	0.13	0.20	0.25	0.31	0.40
40	0.28	0.41	0.54	0.66	0.79	0.15	0.23	0.28	0.33	0.43
45	0.30	0.43	0.55	0.68	0.80	0.16	0.25	0.30	0.36	0.46
50	0.32	0.45	0.57	0.70	0.82	0.18	0.27	0.32	0.38	0.48
55	0.34	0.47	0.60	0.72	0.85	0.20	0.29	0.35	0.41	0.51
60	0.36	0.49	0.62	0.75	0.87	0.23	0.32	0.37	0.43	0.53

Table S7. Centiles for trunk fat percentage by age in Chinese males and females aged 3-60 years.

Age, yrs	Male					Female				
	5th	25th	50th	75th	95th	5th	25th	50th	75th	95th
3	28.8	32.0	34.4	37.0	42.0	28.5	31.4	33.5	35.7	39.3
4	29.0	32.1	34.5	37.2	42.1	28.7	31.6	33.8	36.1	39.8
5	29.1	32.3	34.7	37.4	42.3	28.9	31.9	34.1	36.5	40.3
6	29.3	32.5	34.9	37.6	42.5	29.1	32.2	34.5	36.9	40.9
7	29.5	32.7	35.1	37.9	42.8	29.3	32.5	34.8	37.4	41.4
8	29.7	32.9	35.4	38.2	43.2	29.6	32.9	35.3	37.9	42.1
9	30.0	33.3	35.8	38.6	43.7	30.0	33.4	35.8	38.5	42.8
10	30.4	33.7	36.2	39.1	44.2	30.5	33.9	36.4	39.1	43.5
11	30.9	34.2	36.7	39.6	44.7	31.2	34.6	37.1	39.8	44.2
12	31.5	34.8	37.3	40.2	45.2	31.8	35.3	37.8	40.5	44.9
13	32.3	35.5	38.0	40.8	45.7	32.5	36.0	38.5	41.2	45.6
14	33.1	36.3	38.7	41.5	46.3	33.2	36.7	39.2	41.9	46.2
15	33.9	37.1	39.6	42.3	47.0	33.9	37.3	39.8	42.5	46.9
16	34.8	38.0	40.4	43.1	47.9	34.4	37.9	40.4	43.1	47.4
17	35.5	38.9	41.3	44.0	48.8	34.8	38.3	40.8	43.6	48.0
18	36.2	39.7	42.1	44.8	49.9	35.1	38.6	41.2	44.0	48.4
19	36.6	40.4	42.9	45.7	51.2	35.3	38.9	41.5	44.3	48.8
20	37.0	41.0	43.6	46.5	52.5	35.4	39.0	41.7	44.5	49.1
25	39.0	44.2	47.1	50.4	58.6	35.7	39.7	42.6	45.7	50.9
30	43.2	47.6	50.6	54.0	60.6	36.8	41.1	44.3	47.7	53.2
35	46.0	50.0	53.0	56.4	62.2	38.2	42.6	45.9	49.4	55.1
40	46.8	50.7	53.7	56.9	62.5	39.0	43.4	46.6	50.0	55.7
45	46.5	50.3	53.1	56.2	61.6	39.5	43.8	46.9	50.3	55.8
50	45.8	49.6	52.4	55.4	60.7	40.1	44.3	47.4	50.8	56.2
55	45.1	49.1	52.0	55.0	60.7	41.1	45.2	48.2	51.5	56.7
60	44.6	49.0	51.9	55.1	61.4	42.2	46.1	49.0	52.1	56.9

Table S8. Centiles for leg fat percentage by age in Chinese males and females aged 3-60 years.

Age, yrs	Male					Female				
	5th	25th	50th	75th	95th	5th	25th	50th	75th	95th
3	30.5	36.4	38.7	41.0	46.6	35.5	38.5	40.5	42.3	45.0
4	32.0	36.9	39.2	41.4	45.9	35.8	38.9	40.8	42.7	45.5
5	33.1	37.4	39.7	41.8	45.8	36.1	39.2	41.2	43.2	46.0
6	34.0	37.9	40.1	42.3	45.9	36.3	39.5	41.6	43.6	46.5
7	34.6	38.3	40.5	42.7	46.2	36.5	39.8	41.9	44.0	46.9
8	35.0	38.7	40.9	43.1	46.4	36.7	40.1	42.2	44.3	47.3
9	35.4	38.9	41.2	43.4	46.7	36.8	40.3	42.4	44.6	47.6
10	35.6	39.1	41.4	43.6	46.9	36.8	40.4	42.6	44.7	47.9
11	35.6	39.1	41.4	43.7	46.9	36.8	40.4	42.6	44.8	48.0
12	35.5	39.1	41.4	43.6	46.9	36.7	40.3	42.6	44.8	48.0
13	35.3	38.8	41.1	43.4	46.6	36.5	40.1	42.5	44.7	48.0
14	35.0	38.4	40.7	43.0	46.2	36.2	39.9	42.3	44.5	47.8
15	34.4	37.9	40.2	42.5	45.7	35.9	39.6	42.0	44.3	47.6
16	33.8	37.3	39.6	41.9	45.1	35.6	39.4	41.7	44.0	47.4
17	33.2	36.7	39.0	41.2	44.4	35.2	39.1	41.5	43.8	47.1
18	32.5	36.0	38.3	40.6	43.8	34.9	38.8	41.2	43.6	46.9
19	31.7	35.3	37.7	40.0	43.2	34.7	38.6	41.0	43.4	46.8
20	31.0	34.7	37.0	39.4	42.7	34.4	38.4	40.9	43.3	46.7
25	27.8	31.7	34.3	36.8	40.3	33.3	37.5	40.1	42.6	46.2
30	25.0	29.1	31.8	34.4	38.0	31.5	35.9	38.6	41.2	45.0
35	23.5	27.4	30.0	32.5	36.0	29.7	34.3	37.1	39.8	43.6
40	23.2	26.9	29.4	31.7	35.1	28.4	33.2	36.2	39.0	43.0
45	23.7	27.2	29.5	31.8	34.9	27.2	32.4	35.5	38.5	42.6
50	24.2	27.6	29.8	32.0	35.0	25.9	31.4	34.7	37.8	42.1
55	24.1	27.6	29.8	31.9	35.0	24.5	30.2	33.7	36.9	41.4
60	22.9	27.2	29.5	31.7	35.6	23.0	29.1	32.7	36.1	40.8

Table S9. Centiles for android fat percentage by age in Chinese males and females aged 3-60 years.

Age, yrs	Male					Female				
	5th	25th	50th	75th	95th	5th	25th	50th	75th	95th
3	3.8	4.4	4.9	5.4	6.3	3.9	4.5	5.0	5.5	6.4
4	3.8	4.4	4.9	5.5	6.4	3.9	4.6	5.0	5.6	6.5
5	3.8	4.5	5.0	5.5	6.5	4.0	4.6	5.1	5.7	6.7
6	3.9	4.5	5.0	5.6	6.6	4.0	4.7	5.2	5.8	6.9
7	3.9	4.5	5.1	5.7	6.7	4.1	4.8	5.3	5.9	7.1
8	3.9	4.6	5.1	5.7	6.8	4.1	4.8	5.4	6.1	7.2
9	4.0	4.6	5.2	5.8	6.9	4.2	4.9	5.5	6.2	7.4
10	4.0	4.7	5.3	5.9	7.0	4.2	5.0	5.6	6.3	7.6
11	4.1	4.8	5.3	6.0	7.1	4.3	5.1	5.7	6.4	7.7
12	4.2	4.9	5.4	6.1	7.1	4.4	5.2	5.8	6.5	7.9
13	4.3	5.0	5.5	6.1	7.2	4.5	5.3	5.9	6.7	8.0
14	4.4	5.1	5.6	6.2	7.3	4.6	5.4	6.0	6.8	8.1
15	4.5	5.2	5.7	6.3	7.3	4.7	5.5	6.2	6.9	8.2
16	4.6	5.2	5.8	6.4	7.4	4.8	5.6	6.3	7.1	8.4
17	4.6	5.3	5.8	6.4	7.5	4.9	5.8	6.5	7.2	8.6
18	4.7	5.4	5.9	6.5	7.5	5.1	5.9	6.6	7.4	8.8
19	4.8	5.4	6.0	6.6	7.6	5.2	6.1	6.8	7.5	9.0
20	4.8	5.5	6.0	6.6	7.6	5.3	6.3	7.0	7.7	9.2
25	5.0	5.7	6.3	6.9	8.0	6.1	7.1	7.8	8.6	10.2
30	5.2	6.0	6.6	7.3	8.6	6.8	7.9	8.6	9.5	11.1
35	5.4	6.2	6.9	7.7	9.0	7.3	8.4	9.1	10.0	11.6
40	5.4	6.3	7.0	7.8	9.1	7.5	8.5	9.3	10.2	11.7
45	5.5	6.4	7.1	7.8	9.1	7.5	8.5	9.3	10.1	11.6
50	5.7	6.5	7.2	8.0	9.3	7.4	8.4	9.2	10.0	11.5
55	5.9	6.8	7.5	8.2	9.5	7.3	8.4	9.2	10.0	11.6
60	6.2	7.1	7.7	8.5	9.7	7.2	8.4	9.2	10.1	11.9

Table 10. Centiles for gynoid fat percentage by age in Chinese males and females aged 3-60 years.

Age, yrs	Male					Female				
	5th	25th	50th	75th	95th	5th	25th	50th	75th	95th
3	14.2	15.8	16.8	17.7	19.1	14.6	15.9	16.7	17.5	18.7
4	14.2	15.7	16.7	17.7	19.1	14.7	16.0	16.8	17.7	18.9
5	14.2	15.7	16.7	17.7	19.1	14.7	16.1	17.0	17.8	19.0
6	14.1	15.7	16.7	17.7	19.0	14.7	16.2	17.1	18.0	19.2
7	14.1	15.7	16.7	17.6	19.0	14.8	16.3	17.2	18.1	19.4
8	14.1	15.7	16.7	17.6	19.0	14.8	16.4	17.3	18.3	19.6
9	14.1	15.7	16.7	17.7	19.0	14.9	16.5	17.5	18.5	19.9
10	14.1	15.7	16.7	17.7	19.1	15.0	16.7	17.7	18.7	20.1
11	14.1	15.8	16.8	17.8	19.2	15.2	16.8	17.9	18.9	20.4
12	14.2	15.9	16.9	17.9	19.3	15.4	17.1	18.1	19.2	20.7
13	14.4	16.0	17.0	18.0	19.4	15.6	17.3	18.4	19.4	20.9
14	14.5	16.1	17.1	18.1	19.5	15.8	17.5	18.6	19.7	21.2
15	14.6	16.2	17.2	18.2	19.5	16.0	17.7	18.8	19.9	21.4
16	14.7	16.3	17.3	18.2	19.6	16.1	17.8	18.9	20.0	21.5
17	14.8	16.3	17.3	18.2	19.6	16.2	17.9	19.1	20.1	21.6
18	14.8	16.3	17.3	18.2	19.6	16.3	18.0	19.1	20.2	21.7
19	14.8	16.3	17.3	18.2	19.5	16.4	18.1	19.2	20.3	21.8
20	14.7	16.2	17.2	18.1	19.5	16.4	18.1	19.3	20.3	21.8
25	14.0	15.6	16.7	17.6	19.1	16.2	18.0	19.2	20.3	21.9
30	13.1	14.8	15.9	16.9	18.4	15.5	17.5	18.7	19.9	21.6
35	12.5	14.2	15.2	16.3	17.7	14.7	16.8	18.2	19.5	21.3
40	12.2	13.9	15.0	16.0	17.4	14.2	16.4	17.8	19.2	21.0
45	12.2	13.9	15.0	16.0	17.4	13.9	16.1	17.6	19.0	20.9
50	12.2	13.9	15.0	16.0	17.5	13.6	15.9	17.3	18.7	20.6
55	12.2	13.9	14.9	15.9	17.4	13.5	15.7	17.0	18.3	20.1
60	12.1	13.8	14.8	15.8	17.2	13.4	15.4	16.7	18.0	19.7

Table S11. Centiles for visceral fat percentage by age in Chinese males and females aged 3-60 years.

Age, yrs	Male					Female				
	5th	25th	50th	75th	95th	5th	25th	50th	75th	95th
3	2.3	2.7	2.9	3.2	3.6	0.3	0.7	1.1	1.4	2.1
4	2.1	2.5	2.8	3.1	3.5	0.3	0.7	1.1	1.4	2.0
5	2.0	2.4	2.7	2.9	3.3	0.3	0.7	1.1	1.4	2.0
6	1.8	2.3	2.5	2.8	3.2	0.3	0.8	1.1	1.4	1.9
7	1.7	2.1	2.4	2.7	3.1	0.4	0.8	1.1	1.4	1.9
8	1.6	2.0	2.3	2.6	3.0	0.4	0.8	1.1	1.4	1.9
9	1.4	1.9	2.2	2.5	2.9	0.4	0.8	1.0	1.3	1.8
10	1.4	1.8	2.1	2.4	2.8	0.4	0.8	1.0	1.3	1.8
11	1.3	1.7	2.0	2.3	2.7	0.5	0.8	1.1	1.3	1.7
12	1.2	1.7	1.9	2.2	2.6	0.5	0.8	1.1	1.3	1.7
13	1.2	1.6	1.9	2.1	2.5	0.5	0.8	1.1	1.3	1.7
14	1.1	1.6	1.8	2.1	2.5	0.6	0.9	1.1	1.3	1.7
15	1.1	1.5	1.8	2.1	2.4	0.6	0.9	1.1	1.3	1.7
16	1.1	1.5	1.8	2.0	2.4	0.6	0.9	1.1	1.3	1.7
17	1.1	1.5	1.8	2.0	2.4	0.6	0.9	1.1	1.3	1.7
18	1.1	1.5	1.8	2.1	2.5	0.6	0.9	1.1	1.3	1.7
19	1.1	1.6	1.8	2.1	2.5	0.6	0.9	1.1	1.3	1.7
20	1.1	1.6	1.8	2.1	2.5	0.6	0.9	1.1	1.4	1.7
25	1.1	1.7	2.0	2.3	2.9	0.6	1.0	1.2	1.4	1.8
30	1.2	1.8	2.2	2.5	3.1	0.7	1.1	1.3	1.6	2.1
35	1.3	1.9	2.3	2.7	3.2	0.7	1.2	1.5	1.8	2.3
40	1.5	2.1	2.4	2.8	3.3	0.8	1.3	1.6	1.9	2.4
45	1.6	2.2	2.5	2.9	3.4	0.9	1.4	1.7	2.0	2.6
50	1.8	2.3	2.7	3.0	3.5	1.1	1.5	1.9	2.2	2.8
55	2.0	2.5	2.8	3.1	3.6	1.2	1.7	2.0	2.4	2.9
60	2.1	2.6	3.0	3.3	3.8	1.4	1.9	2.2	2.5	3.0

Table 12. Centiles for subcutaneous fat percentage by age in Chinese males and females aged 3-60 years.

Age, yrs	Male					Female				
	5th	25th	50th	75th	95th	5th	25th	50th	75th	95th
3	3.2	3.9	4.2	4.6	5.7	7.9	9.0	9.8	10.6	12.0
4	3.1	3.8	4.2	4.6	5.8	7.6	8.6	9.4	10.1	11.5
5	3.0	3.7	4.1	4.7	5.9	7.2	8.3	9.0	9.7	11.0
6	2.8	3.5	4.1	4.7	6.0	6.9	7.9	8.6	9.3	10.5
7	2.7	3.4	4.0	4.7	6.2	6.6	7.5	8.2	8.8	10.0
8	2.6	3.3	4.0	4.8	6.2	6.3	7.2	7.8	8.4	9.5
9	2.6	3.2	3.9	4.8	6.3	6.0	6.8	7.4	8.0	9.0
10	2.5	3.1	3.8	4.8	6.3	5.7	6.5	7.0	7.6	8.6
11	2.4	3.0	3.8	4.8	6.2	5.4	6.2	6.7	7.2	8.2
12	2.3	2.9	3.7	4.7	6.2	5.2	5.9	6.4	6.9	7.8
13	2.2	2.8	3.6	4.6	6.0	5.0	5.7	6.2	6.7	7.5
14	2.2	2.8	3.5	4.5	5.9	4.8	5.5	6.0	6.5	7.3
15	2.2	2.7	3.5	4.4	5.8	4.7	5.4	5.8	6.3	7.1
16	2.2	2.7	3.4	4.4	5.7	4.6	5.3	5.7	6.2	7.0
17	2.2	2.7	3.4	4.4	5.7	4.6	5.2	5.7	6.1	6.9
18	2.2	2.8	3.5	4.4	5.6	4.6	5.2	5.6	6.1	6.9
19	2.3	2.8	3.5	4.4	5.6	4.6	5.2	5.6	6.1	6.9
20	2.4	2.9	3.6	4.4	5.6	4.6	5.2	5.6	6.1	6.9
25	2.8	3.4	3.9	4.6	5.8	4.6	5.3	5.7	6.2	7.0
30	3.2	3.8	4.3	4.9	6.0	4.7	5.3	5.8	6.2	7.0
35	3.4	4.1	4.5	5.1	6.2	4.6	5.3	5.7	6.2	7.0
40	3.5	4.2	4.6	5.2	6.2	4.6	5.2	5.7	6.1	6.9
45	3.6	4.2	4.6	5.1	6.1	4.6	5.2	5.7	6.1	6.9
50	3.6	4.2	4.6	5.1	6.1	4.6	5.3	5.7	6.2	7.0
55	3.6	4.3	4.6	5.0	6.1	4.7	5.4	5.8	6.3	7.1
60	3.6	4.3	4.7	5.1	6.1	4.8	5.4	5.9	6.3	7.1

Table S13. Covariates-adjusted total fat mass (FM), fat mass index (FMI) and body fat percentage (BF%) among Chinese, US non-Hispanic White, non-Hispanic Black and Mexican American across different stages of life*

Whole body fat measures	Middle Childhood (8-11y)		Adolescence (12-19y)		Early adulthood (20-39y)		Middle adulthood (40-60y)	
	Male	Female	Male	Female	Male	Female	Male	Female
FM, kg								
Chinese population	9.9 (0.2)	10.2 (0.3)	14.3 (0.2)	16.5 (0.2)	17.5 (0.7)	19.7 (0.7)	17.6 (0.8)	21.5 (0.6)
US non-Hispanic White	12.4 (0.3)	14.0 (0.3)	18.4 (0.6)	23.6 (0.5)	24.1 (0.4)	29.1 (0.5)	27.0 (0.4)	31.0 (0.5)
US non-Hispanic Black	12.1 (0.4)	14.3 (0.4)	17.8 (0.6)	22.4 (0.6)	21.6 (0.6)	32.3 (0.6)	25.3 (0.5)	37.1 (0.5)
US Mexican American	14.8 (0.4)	15.2 (0.4)	21.2 (0.7)	25.4 (0.6)	25.9 (0.7)	31.3 (0.7)	26.2 (0.6)	32.3 (0.7)
P value (Chinese vs White)	<0.001	<0.001	<0.001	<0.001	<0.001	<0.001	<0.001	<0.001
P value (Chinese vs Black)	0.001	<0.001	<0.001	<0.001	0.008	<0.001	<0.001	<0.001
P value (Chinese vs Mexican)	<0.001	<0.001	<0.001	<0.001	<0.001	<0.001	<0.001	<0.001
P value (White vs Black)	<0.001	0.006	<0.001	0.001	<0.001	<0.001	0.208	<0.001
P value (White vs Mexican)	<0.001	0.003	<0.001	<0.001	<0.001	0.691	0.001	0.061
P value (Black vs Mexican)	0.806	0.994	0.911	0.499	0.001	<0.001	0.049	<0.001
FMI, kg/m^2								
Chinese population	5.0 (0.1)	5.1 (0.1)	4.9 (0.1)	6.4 (0.1)	5.9 (0.2)	7.8 (0.3)	6.1 (0.2)	8.6 (0.2)
US non-Hispanic White	5.9 (0.1)	6.7 (0.1)	6.2 (0.2)	8.9 (0.2)	7.7 (0.1)	10.7 (0.2)	8.6 (0.1)	11.7 (0.2)

US non-Hispanic Black	5.7 (0.2)	6.6 (0.2)	6.0 (0.2)	8.5 (0.2)	6.9 (0.2)	12 (0.2)	8.1 (0.2)	14.0 (0.2)
US Mexican American	7.1 (0.2)	7.4 (0.2)	7.3 (0.2)	10.0 (0.2)	8.7 (0.2)	12.4 (0.3)	9.1 (0.2)	13.1 (0.3)
P value (Chinese vs White)	<0.001	<0.001	<0.001	<0.001	<0.001	<0.001	<0.001	<0.001
P value (Chinese vs Black)	0.001	<0.001	<0.001	<0.001	0.008	<0.001	<0.001	<0.001
P value (Chinese vs Mexican)	<0.001	<0.001	<0.001	<0.001	<0.001	<0.001	<0.001	<0.001
P value (White vs Black)	<0.001	0.006	<0.001	0.001	<0.001	<0.001	0.208	<0.001
P value (White vs Mexican)	<0.001	0.003	<0.001	<0.001	<0.001	0.691	0.001	0.061
P value (Black vs Mexican)	0.806	0.994	0.911	0.499	0.001	<0.001	0.049	<0.001
BF%								
Chinese population	26.9 (0.3)	28.9 (0.3)	22.2 (0.2)	30.7 (0.2)	23.1 (0.4)	32.2 (0.4)	24.4 (0.4)	34.1 (0.3)
US non-Hispanic White	29.3 (0.4)	32.5 (0.4)	24.3 (0.5)	33.8 (0.4)	26.3 (0.3)	36.9 (0.3)	28.5 (0.2)	39.1 (0.3)
US non-Hispanic Black	27.2 (0.5)	30.6 (0.4)	22.2 (0.5)	32.0 (0.5)	23.3 (0.3)	37.5 (0.4)	26.4 (0.3)	40.9 (0.3)
US Mexican American	32.6 (0.5)	34.9 (0.4)	26.6 (0.6)	36.1 (0.5)	28.4 (0.4)	39.3 (0.4)	29.0 (0.4)	41.0 (0.4)
P value (Chinese vs White)	<0.001	<0.001	<0.001	<0.001	<0.001	<0.001	<0.001	<0.001
P value (Chinese vs Black)	0.001	<0.001	<0.001	<0.001	0.008	<0.001	<0.001	<0.001
P value (Chinese vs Mexican)	<0.001	<0.001	<0.001	<0.001	<0.001	<0.001	<0.001	<0.001
P value (White vs Black)	<0.001	0.006	<0.001	0.001	<0.001	<0.001	0.208	<0.001
P value (White vs Mexican)	<0.001	0.003	<0.001	<0.001	<0.001	0.691	0.001	0.061
P value (Black vs Mexican)	0.806	0.994	0.911	0.499	0.001	<0.001	0.049	<0.001

a. Comparisons were conducted using analysis of covariance adjusted for age, socioeconomic status, physical activity, sugary drink consumption and sleep duration.

Table S14. Covariates-adjusted trunk-to-leg fat ratio (TLR), android-to-gynoid fat ratio (AGR), and visceral-to-subcutaneous fat ratio (VSR) among Chinese, US non-Hispanic White, non-Hispanic Black and Mexican American across different stages of life*.

Fat distribution measures	Middle Childhood (8-11y)		Adolescence (12-19y)		Early adulthood (20-39y)		Middle adulthood (40-60y)	
	Male	Female	Male	Female	Male	Female	Male	Female
TLR								
Chinese population	0.89 (0.01)	0.86 (0.01)	1.04 (0)	0.98 (0.01)	1.4 (0.02)	1.17 (0.02)	1.69 (0.03)	1.51 (0.02)
US non-Hispanic White	0.80 (0.01)	0.83 (0.01)	0.97 (0.01)	0.91 (0.01)	1.32 (0.01)	1.12 (0.01)	1.70 (0.02)	1.32 (0.02)
US non-Hispanic Black	0.74 (0.01)	0.74 (0.01)	0.86 (0.01)	0.82 (0.02)	1.12 (0.02)	1.04 (0.02)	1.44 (0.02)	1.22 (0.02)
US Mexican American	0.88 (0.01)	0.92 (0.01)	1.08 (0.02)	1.11 (0.02)	1.54 (0.02)	1.31 (0.02)	1.89 (0.02)	1.51 (0.03)
P value (Chinese vs White)	<0.001	0.096	<0.001	<0.001	0.004	0.126	1.000	<0.001
P value (Chinese vs Black)	<0.001	<0.001	<0.001	<0.001	<0.001	<0.001	<0.001	<0.001
P value (Chinese vs Mexican)	0.998	0.001	0.122	<0.001	<0.001	<0.001	<0.001	0.999
P value (White vs Black)	<0.001	<0.001	<0.001	<0.001	<0.001	<0.001	<0.001	<0.001
P value (White vs Mexican)	<0.001	<0.001	<0.001	<0.001	<0.001	<0.001	<0.001	<0.001
P value (Black vs Mexican)	<0.001	<0.001	<0.001	<0.001	<0.001	0.001	<0.001	0.003
AGR								
Chinese population	0.35 (0.00)	0.31 (0.00)	0.37 (0.00)	0.31 (0.00)	0.50 (0.01)	0.38 (0.01)	0.59 (0.01)	0.46 (0.01)
US non-Hispanic White	0.34 (0.00)	0.33 (0.00)	0.38 (0.01)	0.32 (0.01)	0.49 (0.01)	0.40 (0.01)	0.65 (0.01)	0.47 (0.01)

US non-Hispanic Black	0.31 (0.01)	0.31 (0.01)	0.35 (0.01)	0.31 (0.01)	0.43 (0.01)	0.39 (0.01)	0.57 (0.01)	0.47 (0.01)
US Mexican American	0.39 (0.01)	0.38 (0.01)	0.42 (0.01)	0.39 (0.01)	0.58 (0.01)	0.47 (0.01)	0.71 (0.01)	0.53 (0.01)
P value (Chinese vs White)	0.779	0.003	0.731	0.207	0.895	0.019	<0.001	0.890
P value (Chinese vs Black)	<0.001	0.937	0.027	0.886	<0.001	0.616	0.729	0.928
P value (Chinese vs Mexican)	<0.001	<0.001	<0.001	<0.001	<0.001	<0.001	<0.001	<0.001
P value (White vs Black)	<0.001	<0.001	<0.001	<0.001	<0.001	<0.001	<0.001	<0.001
P value (White vs Mexican)	<0.001	<0.001	<0.001	<0.001	<0.001	<0.001	<0.001	<0.001
P value (Black vs Mexican)	0.001	0.002	0.010	0.164	<0.001	0.493	<0.001	1.000
VSR								
Chinese population	0.59 (0.01)	0.15 (0.00)	0.57 (0.01)	0.19 (0.00)	0.55 (0.01)	0.24 (0.00)	0.62 (0.01)	0.35 (0.01)
US non-Hispanic White	0.55 (0.01)	0.15 (0.00)	0.45 (0.01)	0.14 (0.00)	0.40 (0.01)	0.19 (0.00)	0.50 (0.01)	0.28 (0.00)
US non-Hispanic Black	0.60 (0.01)	0.12 (0.00)	0.49 (0.02)	0.12 (0.01)	0.43 (0.01)	0.15 (0.00)	0.44 (0.01)	0.22 (0.01)
US Mexican American	0.45 (0.02)	0.17 (0.00)	0.37 (0.02)	0.16 (0.01)	0.35 (0.01)	0.22 (0.00)	0.51 (0.01)	0.31 (0.01)
P value (Chinese vs White)	0.187	1.000	<0.001	<0.001	<0.001	<0.001	<0.001	<0.001
P value (Chinese vs Black)	0.955	<0.001	<0.001	<0.001	<0.001	<0.001	<0.001	<0.001
P value (Chinese vs Mexican)	<0.001	<0.001	<0.001	<0.001	<0.001	0.001	<0.001	<0.001
P value (White vs Black)	<0.001	0.001	0.001	0.105	0.002	<0.001	0.996	<0.001
P value (White vs Mexican)	<0.001	<0.001	<0.001	<0.001	<0.001	<0.001	<0.001	<0.001
P value (Black vs Mexican)	0.098	<0.001	0.264	0.001	0.063	<0.001	<0.001	<0.001

*. Comparisons were conducted using analysis of covariance adjusted for age, socioeconomic status, physical activity, sugary drink consumption and sleep duration.
